# Peripheral Expression of Mutant Huntingtin is a Critical Determinant of Weight Loss and Metabolic Disturbances in Huntington’s Disease

**DOI:** 10.1038/s41598-019-46470-8

**Published:** 2019-07-12

**Authors:** Priya Lakra, Kumari Aditi, Namita Agrawal

**Affiliations:** Department of Zoology, University of Delhi, Delhi, 110007 India

**Keywords:** Apoptosis, Protein aggregation, Huntington's disease, Fat metabolism, Feeding behaviour

## Abstract

Deteriorating weight loss in patients with Huntington’s disease (HD) is a complicated peripheral manifestation and the cause remains poorly understood. Studies suggest that body weight strongly influences the clinical progression rate of HD and thereby offers a valuable target for therapeutic interventions. Mutant huntingtin (mHTT) is ubiquitously expressed and could induce toxicity by directly acting in the peripheral tissues. We investigated the effects of selective expression of mHTT exon1 in fat body (FB; functionally equivalent to human adipose tissue and liver) using transgenic *Drosophila*. We find that FB-autonomous expression of mHTT exon1 is intrinsically toxic and causes chronic weight loss in the flies despite progressive hyperphagia, and early adult death. Moreover, flies exhibit loss of intracellular lipid stores, and decline in the systemic levels of lipids and carbohydrates which aggravates over time, representing metabolic defects. At the cellular level, besides impairment, cell death also occurs with the formation of mHTT aggregates in the FB. These findings indicate that FB-autonomous expression of mHTT alone is sufficient to cause metabolic abnormalities and emaciation *in vivo* without any neurodegenerative cues.

## Introduction

Huntington’s disease (HD) is a chronic, progressive neurodegenerative disorder with no known cure. It is caused by the polymorphic CAG repeat expansion in the exon1 region of the huntingtin (*HTT*) gene^1^ which is expressed ubiquitously in human tissues^2–4^ and is translated into a mutated form of the huntingtin protein (mHTT), which harbours a polyglutamine expansion. HD has always been characterized as primarily a neurodegenerative disorder; however, contrary to the traditional thinking, recent studies suggest it may be a systemic disorder. In patients with HD, besides the hallmark neurological manifestations, unintended progressive weight loss is a major peripheral manifestation which further complicates this disorder^5–11^. Weight loss is reported to start early in pre-symptomatic carriers^9,12^ and becomes malignant in the later stages of the disease^5,7,13^. Recent studies are strengthening the hypothesis that body weight is an important factor which influences the clinical progression rate of HD as higher body mass indices have been associated with slower rate of the disease progression^14,15^. Besides being a robust predictor in disease progression, it also worsens the quality of life of the patients^16,17^. Patients experience weight loss despite having normal^13,18^ to high caloric intake^10,12^. Surprisingly, weight loss in HD doesn’t seem to correlate with chorea since it is present in early stages of HD^9^ where chorea is minimal and becomes severe in the later stages when patients are usually rigid and bed-ridden^7,8,19,20^. Besides weight loss, metabolic abnormalities characterized by increased appetite and higher food intake^10,12^, nutritional deficiencies^13,21,22^, glucose imbalance and insulin resistance^23–25^, hepatic dysfunction^26–29^, and altered leptin levels^30,31^ are frequently reported in patients with HD. Subsequently, several studies have suggested that weight loss in HD could be attributed to the metabolic disturbances^12,25,30–34^.

Ever since the characterization of weight loss and metabolic abnormalities in HD, most studies describe them as a secondary effect of neurodegeneration but due to the poor correlation between neurodegeneration and weight loss, the underlying pathophysiology is yet unclear^34,35^. Nevertheless, the cause of deteriorating weight loss may lie beyond the neurons since mutant *HTT* is expressed in an array of peripheral tissues. Several transgenic HD models expressing the exon1 fragment of mutant *HTT* including R6/2 mice^33,34,36,37^, *Drosophila*^38^, recapitulate the weight loss in HD and indicate abnormalities in the peripheral tissues influencing metabolic homeostasis like adipose tissue^36,38,39^ and gastro-intestinal tract^33^. However, whether this weight loss and altered metabolism are primarily due to cell-autonomous effects of mHTT in peripheral tissues or are an indirect effect of widespread neurodegeneration or general malaise remains inconclusive. In fact, a growing body of evidence describes peripheral tissue abnormalities such as adipose tissue, liver, skeletal muscle, gut and pancreas, in patients with HD^40–43^. Interestingly, weight loss in patients is accompanied by substantial loss of body fat stores^6,10,44^ and a recent study revealed that the fat-free mass was not significantly different between control and HD subjects while body mass index (BMI) was lower in HD subjects^45^. Another study has suggested adipocyte abnormalities in early-stage HD patients which may worsen with disease progression and could contribute to weight loss^31^. Moreover, mHTT is reported to cause wide-scale alterations in several cellular processes which are crucial to all cell types like transcriptional deregulation, mitochondrial dysfunction, impairment in autophagic processes, ubiquitin-proteasome system, and vesicle transport^46–48^; therefore, presence of mHTT even in peripheral tissues may cause considerable damage in HD setting.

Here, we hypothesize that the weight loss and metabolic dysfunction in HD can be primarily caused by direct detrimental effects engendered by mHTT in adipose tissue independent of neurodegeneration. Over the past few decades, *Drosophila* models have provided significant insights in the study of human metabolic disorders like diabetes, insulin resistance, and metabolic syndrome^49–51^ given the amply analogy with mammalian metabolic organ systems and various well-conserved metabolic and energy homeostasis pathways^51,52^. Furthermore, *Drosophila* provides an excellent system for spatiotemporal expression of genes and studying their direct effects *in vivo*. To test this hypothesis, we employed the *Drosophila* fat body (FB) as it is functionally equivalent to human adipose tissue and liver^53^.The FB is a key dynamic organ playing an indispensable role in maintaining metabolic homeostasis and systemic growth of the organism. It performs multiple metabolic functions including energy storage and mobilization. It also functions as a nutrient sensor and in turn couples nutrient status to energy demands of the flies^54,55^. By selectively expressing human mHTT exon1 in the *Drosophila* FB, we find that FB-autonomous expression of human mHTT exon1 is intrinsically toxic and leads to hyperphagic weight loss, metabolic dysfunction and eventual death of the flies. We further find progressive degeneration of FB in these flies with a concomitant accumulation and aggregation of mHTT exon1 peptides in the FB.

## Results

### FB-autonomous expression of mHTT induces weight loss in flies despite higher food intake and eventual death

**Figure 1:**
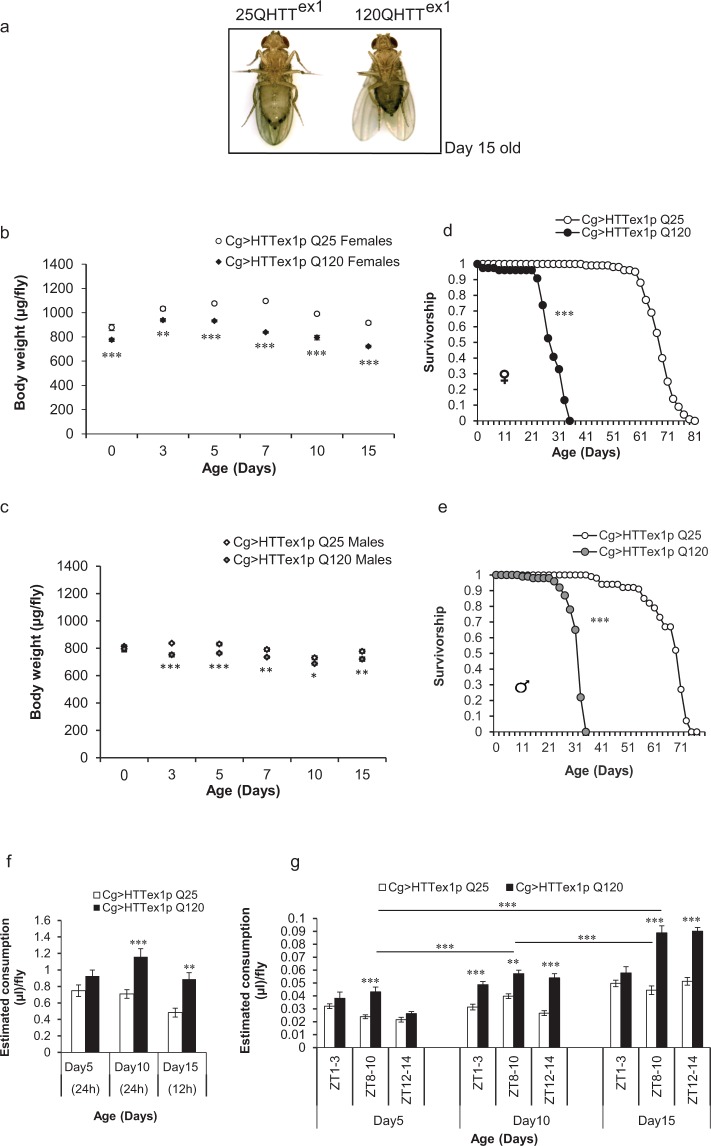
FB-autonomous expression of mHTT exon1 causes chronic weight loss despite higher food intake, and early adult death. Body weights, food intake and lifespan of *ad libitum* fed 25QHTT^ex1^ (Cg > HTTex1p Q25; open labels) and 120QHTT^ex1^ (Cg > HTTex1p Q120; black labels) flies were measured at the indicated ages. **(a)** 120QHTT^ex1^ females become phenotypically lean with shrunken abdomen by day15 posteclosion. **(b)** Age-dependent alterations in body weight occur in 120QHTT^ex1^ females when compared with age-matched 25QHTT^ex1^ females. 120QHTT^ex1^ females are lean and have low body weight at the time of eclosion (day0) which increases by day3, followed by a progressive decrease in body weight throughout the indicated time points in comparison to the age-matched 25QHTT^ex1^. Values represent mean ± SEM. (*n* = 50/genotype/age). **(c)** Age-dependent alterations in body weight occur in 120QHTT^ex1^ males when compared with age-matched 25QHTT^ex1^ males. 120QHTT^ex1^ males have comparable body weight at the time of eclosion (day0), followed by a decrease in body weight throughout the indicated time points in comparison to the age-matched 25QHTT^ex1^. Values represent mean ± SEM. (*n* = 50/genotype/age). **(d)** 120QHTT^ex1^ females show early lethality with a mean lifespan of 28.37 days, while the 25QHTT^ex1^ have a mean lifespan of 68.22 days. (*n* = 100 flies/genotype) **(e)** 120QHTT^ex1^ males show early lethality with a mean lifespan of 31.6 days, while the 25QHTT^ex1^ males have a mean lifespan of 66.58 days. (*n* = 100 flies/genotype) **(f)** *Ad libitum* food intake of 25QHTT^ex1^ and 120QHTT^ex1^ females was assessed by capillary feeder (CAFE) assay at the respective ages. 120QHTT^ex1^ females show higher food intake at day10 and day15 of age compared to the age-matched 25QHTT^ex1^ adults (*n* = 56–65/genotype/age) **(g)** Feeding patterns of 25QHTT^ex1^ and 120QHTT^ex1^ females was monitored at three different Zeitgeber time (ZT) points where adults were fed blue dye labelled food for 2 hours at these time points over 14-hours at the indicated ages. 120QHTT^ex1^ adults develop feeding behaviour defects and consume significantly higher food compared to the age-matched 25QHTT^ex1^. Also, the 120QHTT^ex1^ display progressive hyperphagia. Values represent mean ± SEM. (*n* = 50–60/genotype/age). **P < 0.01; ***P < 0.001.

To shed light on the direct effects of human mHTT on *Drosophila* FB, we examined the effects of targeted expression of HTT exon1 with unexpanded (25Qs, wild-type) and expanded (120Qs, mutant) glutamines in the FB of flies. We expressed the transgenes selectively in the FB of the flies at 25 °C under the control of Cg-GAL4 (see Supplementary Fig. S1 for *Cg* expression profile) since the embryonic stage. Cg > HTTex1p Q25 (referred hereafter as 25QHTT^ex1^) and Cg > HTTex1p Q120 (referred hereafter as 120QHTT^ex1^) flies grown under standard conditions were aged up to 15 days and compared for body weight alterations. We found that 120QHTT^ex1^ flies exhibited declining body weight with ageing and the females became phenotypically lean by day15 with shrunken abdomens (Fig. 1a,b, S2). The reduction in the body weight of 120QHTT^ex1^ females was evident at eclosion (day0) and became severe by day15 (Fig. 1b; two-factor ANOVA; genotype main effect: F(1, 48) = 405.8, *P* < 0.0001; age main effect: F(5, 48) = 66.12, *P* < 0.0001; age × genotype effect: F(5, 48) = 9.945, *P* < 0.0001). The newly eclosed 120QHTT^ex1^ females displayed 11.68% decline in the body weight and this decline increased to 21.17% by day15 of age. Sibling 120QHTT^ex1^ males also showed reduction in body weight overtime. At day0, no significant difference in weight was observed between 120QHTT^ex1^ males and corresponding 25QHTT^ex1^ males. However, day3 posteclosion, the body weight of 120QHTT^ex1^ males began to decline by 10.15% and it remained less as compared to the 25QHTT^ex1^ males throughout the indicated ages (Fig. 1c; two-factor ANOVA; genotype main effect: F(1, 48) = 111.8, *P* < 0.0001; age main effect: F(5, 48) = 33.96, *P* < 0.0001; age × genotype effect: F(5, 48) = 3.174, *P* = 0.0148). We also compared if body weight of 25QHTT^ex1^ is comparable to that of the wild-type *Drosophila melanogaster*. Literature survey displayed that the range of body weight of 3–5 days old wild-type *Drosophila melanogaster* is between 0.8–0.9 mg/fly for males and 1–1.5 mg/fly for females^56–58^ and the body weight of the 25QHTT^ex1^ adults also falls within this range (0.83 mg/fly for male and 1.1 mg/fly for female 3 days posteclosion). Therefore, it suggests that the 25QHTT^ex1^ flies used in this study behave similarly as the non- transgenic controls in terms of their body weight and metabolite profile.

Although we cannot exclude minor variations in the metabolic profile that can occur between 25QHTT^ex1^ and wild-type flies due to the differences in the genetic background as it has been extensively noted that body weight and metabolic homeostasis of an organism are complex phenomenon that depends highly on the genetics of the organism^59,60^. Therefore, in order to minimize such variations in the metabolic profile, we have compared the effect of human HTT exon1 with 25Qs vs. 120Qs in transgenic flies with targeted insertion of foreign transgenes in a common genetic background^61^.

The insect FB is critically involved in the development and growth of the organism^55^. Besides the observed weight loss in 120QHTT^ex1^ flies, we asked whether this targeted expression of mHTT exon1 in the FB has any effect on the viability of the flies. Despite the persistent expression of mHTT exon1 from embryogenesis onwards, the 120QHTT^ex1^ flies eclosed normally with no significant lethality during eclosion (data not shown). Strikingly, the 120QHTT^ex1^ flies showed an early death phenotype. On the contrary, no death occurred in the 25QHTT^ex1^ flies during this time period. 120QHTT^ex1^ females had reduced longevity with a mean lifespan of 28.37 ± 0.69 days (Fig. 1d; Log-rank test (25QHTT^ex1^ females vs. 120QHTT^ex1^ females), *P* = 0.0E + 00). Similarly, sibling males also exhibited reduced longevity with a mean lifespan of 31.60 ± 0.39 days (Fig. 1e, Log-rank test (25QHTT^ex1^ males vs. 120QHTT^ex1^ males), *P* = 0.0E + 00). 25QHTT^ex1^ flies had a lifespan of about 2.5 months (Females, mean lifespan: 68.22 ± 0.61 days; Males, mean lifespan: 66.58 ± 0.88 days) in our hands. The viability profiles indicate that the FB-targeted expression of mHTT exon1 is an insidious event.

To investigate if hypophagia contributes to the observed weight loss in 120QHTT^ex1^ flies, we measured the food intake of 25QHTT^ex1^ and 120QHTT^ex1^ flies at different ages i.e. 5-, 10- and 15-day-old (Fig. 1f,g). To measure *ad libitum* food consumption, we initially employed capillary feeder (CAFE) assay^62^. We found that the food intake of 120QHTT^ex1^ flies tended to be higher at day5 but biological significance was not established. However, food intake of 120QHTT^ex1^ flies was significantly higher at day10 and day15, than age-matched 25QHTT^ex1^ (Fig. 1f; two-factor ANOVA; genotype main effect: F(1, 78) = 33.93, *P* < 0.0001; age main effect: F(2, 78) = 6.052, *P* = 0.0036; age × genotype effect: F(2, 78) = 2.037, *P* = 0.1374). A major limitation of the CAFE assay was the death of 120QHTT^ex1^ flies after 10 days of age when kept beyond 24 hours in the CAFE setup vials which prevented longitudinal assessment of feeding in the same set of flies. Since 120QHTT^ex1^ flies exhibit gradual decline in their body weight and are weaker than the 25QHTT^ex1^ and the CAFE setup requires flies to climb down the micro-capillary and actively hold itself in this upside-down position to feed, therefore, lethargy or any locomotor deficiencies will affect the food intake of these flies. Thereby, to avoid any such variances due to the CAFE assay setup, we also monitored the consumption of solid food by these flies, present on the bottom of the vials, using blue dye feeding assay. At first, groups of females were allowed to feed on standard food supplemented with FD&C blue dye #1 (2% w/v) for 2 hours in the morning (Zeitgeber time (ZT) 1–3), evening (ZT 8–10) and night (ZT 12–14). We found comparable results in the CAFE assay and the blue dye feeding assay where the food intake of 120QHTT^ex1^ flies differed significantly from that of 25QHTT^ex1^ (Fig. 1g; two-factor ANOVA; genotype main effect: F(1, 179) = 194.4456, *P* < 0.0001; age main effect: F(8, 179) = 52.7689, *P* < 0.0001; age × genotype effect: F(8, 179) = 10.7202, *P* < 0.0001). The results of blue dye feeding assay revealed that at day5, 120QHTT^ex1^ flies consumed higher food only at ZT 8–10 while at rest of the time points food consumption was comparable to the corresponding 25QHTT^ex1^. However, at day10 and day15 of age, 120QHTT^ex1^ flies displayed significantly increased feeding compared to the age-matched 25QHTT^ex1^ (Fig. 1g). Interestingly, further analysis revealed that the increase in food intake of 120QHTT^ex1^ flies was progressive with strikingly higher food consumption at day15 (Fig. 1g; Tukey post-hoc test; day5 vs. day10, *P* < 0.0001; day10 vs. day15, *P* < 0.0001; day5 vs. day15, *P* < 0.0001). Thereby, decreased food intake is ruled out as the factor responsible for weight loss and despite having increased food consumption, flies were unable to maintain their body weight. Hence, the specific expression of mutant *HTT* as a transgene throughout the development in the FB of *Drosophila* is associated with severe weight loss with higher food consumption and is toxic to the adult flies; phenotypes reminiscent of that observed in HD patients^5,10,12,15^.

### 120QHTT^ex1^ flies exhibit a decline in lipid levels

**Figure 2:**
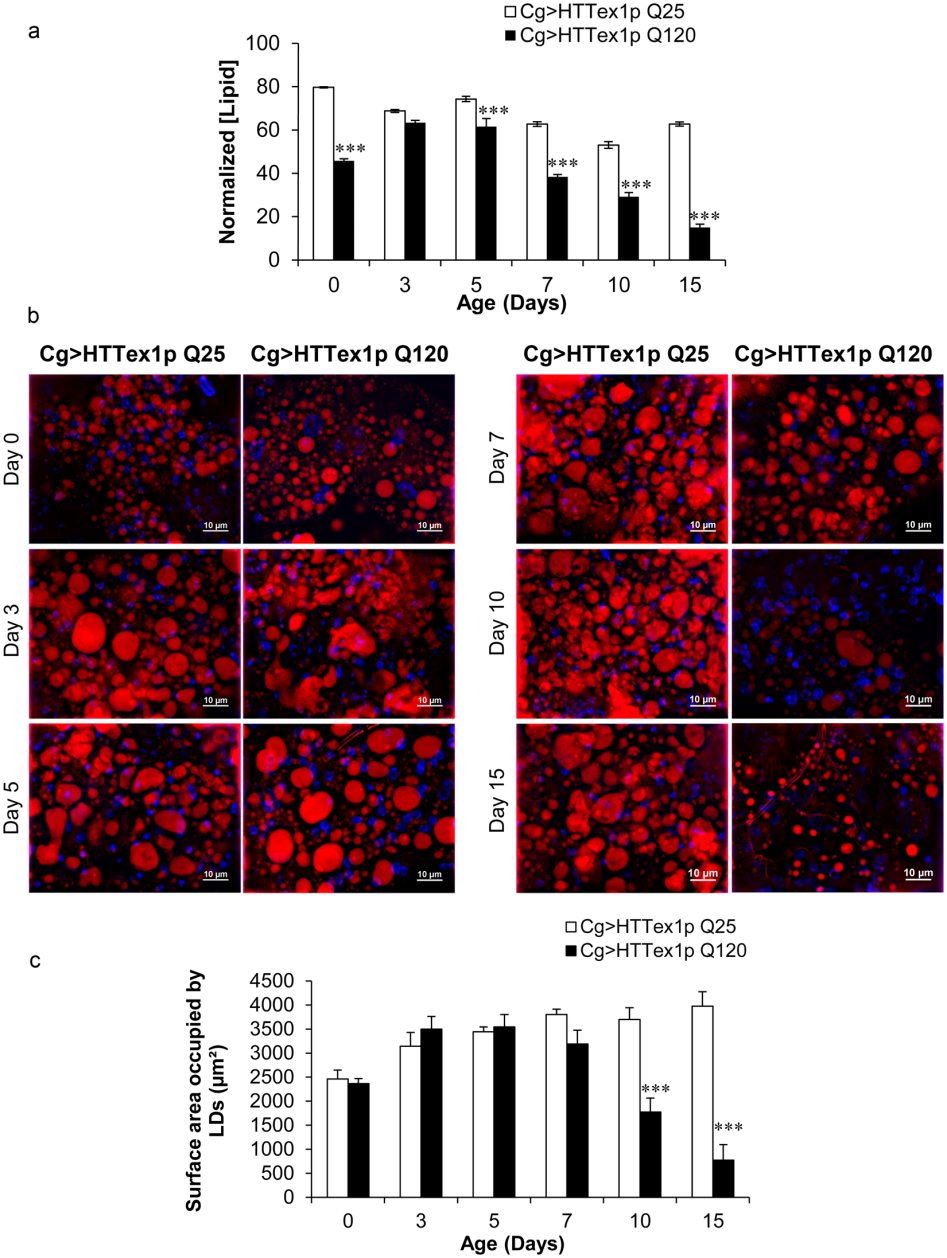
Age-dependent decline in lipid content of 120QHTT^ex1^ adults. (**a**) Whole fly lipid content was measured in 25QHTT^ex1^ and 120QHTT^ex1^ females under *ad libitum* fed condition and normalized with their respective body weight. 120QHTT^ex1^ females exhibit gradual decline in systemic lipid levels compared to their corresponding 25QHTT^ex1^ flies. (*n* = 50/genotype/age) (**b)** Nile red staining of the abdominal FB of 25QHTT^ex1^and 120QHTT^ex1^ females at the indicated ages revealed sparse lipid droplets in 120QHTT^ex1^ females. 120QHTT^ex1^ females display significant reduction in the size of the lipid droplets at the later ages, i.e., day10 and day15 relative to the 25QHTT^ex1^. 25QHTT^ex1^, however, maintain a constant distribution of intracellular lipids droplets throughout these ages. (*n* = 5/genotype/age) Scale bar, 10 µm. (**c)** Quantification of total surface area of lipid droplets in the FB of 25QHTT^ex1^ and 120QHTT^ex1^ females further validated the significant decline in total area occupied by lipid droplets in day10 and day15 old 120QHTT^ex1^ females. Values represent mean ± SEM. ***P < 0.001.

As an initial step towards the understanding of these aggravated weight changes with ageing, we quantified the lipids which represent a major proportion of the stored energy in the FB^54,55^. We measured the systemic levels of ether extractable lipids of the 25QHTT^ex1^ and 120QHTT^ex1^ adults at different ages posteclosion; day0, day3, day5, day7, day10 and day15. On comparison with the age-matched 25QHTT^ex1^, the newly eclosed 120QHTT^ex1^ adults already had considerable lower lipids relative to body weight and further analysis showed that these lipid levels declined progressively over time and became severely low by day15 of age compared to the corresponding 25QHTT^ex1^ (Fig. 2a; two-factor ANOVA; genotype main effect: F(1, 48) = 665.496, *P* < 0.0001; age main effect: F(5, 48) = 119.746, *P* < 0.0001; age × genotype effect: F(5, 48) = 41.0432, *P* < 0.0001). Conclusively, 120QHTT^ex1^ flies displayed a significant decline in the systemic lipid content relative to body weight with ageing while 25QHTT^ex1^ flies maintained the levels of systemic lipids relative to body weight throughout ageing.

The *Drosophila* FB contains a substantial amount of specialized intracellular organelles known as lipid droplets. Lipid droplets store lipids primarily in the form of triglycerides^55^. In line with the systemic depletion of lipids, we measured the lipid droplet distribution in the abdominal FB of adults with ageing. The lipid droplets of the abdominal FB cells were visualised using Nile red staining in both 25QHTT^ex1^ and 120QHTT^ex1^ flies. Adults were analyzed for lipid droplet distribution in the abdominal FB cells at the ages indicated above. Notably, we found a visible depletion of the lipid droplets in the FB cells of 120QHTT^ex1^ flies by day10 (Fig. 2b). Upon quantification, no significant depletion in the lipid droplets was observed until day7 in 120QHTT^ex1^ flies when compared with the age-matched 25QHTT^ex1^. However, the FB of 120QHTT^ex1^ flies had significantly fewer and smaller lipid droplets at day10 and day15 compared with the corresponding 25QHTT^ex1^ flies (Fig. 2c; two-factor ANOVA; genotype main effect: F(1, 48) = 41.02, *P* < 0.0001; age main effect: F(5, 48) = 9.442, *P* < 0.0001; age × genotype effect: F(5, 48) = 16.33, *P* < 0.0001). Altogether, our observations indicate that there is a chronic decline in the whole-body lipid levels along with the substantial depletion of lipids in the FB cells of 120QHTT^ex1^ flies.

### 120QHTT^ex1^ flies exhibit gradual decline in carbohydrates

**Figure 3:**
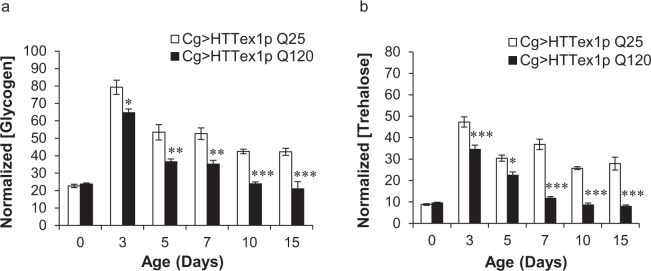
Gradual decline in carbohydrates of 120QHTT^ex1^ adults. (**a)** At the indicated ages, whole fly glycogen content was measured in 25QHTT^ex1^ and 120QHTT^ex1^ females. Glycogen levels decrease progressively after 3 days of age compared to the age-matched 25QHTT^ex1^. (**b)** Whole fly trehalose content was also measured for these flies at the same ages. The levels of trehalose follow the similar pattern and decline progressively after day3 posteclosion in 120QHTT^ex1^ females as compared to the age-matched 25QHTT^ex1^. The levels of glycogen and trehalose were normalized with the body weight of the respective flies. Values represent mean ± SEM. (*n* = 20/genotype/age) *P < 0.05; **P < 0.01; ***P < 0.001.

We sought to determine the effect on other energy biomolecules as well. In *Drosophila*, besides lipids, glycogen is another important energy reserve stored in the FB. Glycogen, stored in the FB, is converted to hemolymph trehalose in response to the animal energy demands^54,55^. Circulating trehalose acts as a homeostatic biomolecule originating from the FB. Thereby, we assessed the levels of these two carbohydrates, namely, glycogen and trehalose in our flies with ageing. We found significant age-dependent alterations in the systemic levels of glycogen as well as trehalose in 120QHTT^ex1^ flies compared to the corresponding 25QHTT^ex1^ flies. At day0, the glycogen levels were comparable in the 120QHTT^ex1^ and 25QHTT^ex1^ flies. The decline in the systemic glycogen levels of 120QHTT^ex1^ flies initiated 3 days posteclosion and the levels declined progressively thereafter (Fig. 3a; two-factor ANOVA; genotype main effect: F(1, 48) = 90.7, *P* < 0.0001; age main effect: F(5, 48) = 82.07, *P* < 0.0001; age × genotype effect: F(5, 48) = 4.517, *P* = 0.0019). Similar pattern was observed in the trehalose levels of the 120QHTT^ex1^ flies. Systemic decline in trehalose levels was evident at day3 and severe at the later ages compared with the age-matched 25QHTT^ex1^ (Fig. 3b; two-factor ANOVA; genotype main effect: F(1, 48) = 217, *P* < 0.0001; age main effect: F(5, 48) = 90.12, *P* < 0.0001; age × genotype effect: F(5, 48) = 16.34, *P* < 0.0001). On the contrary, 25QHTT^ex1^ flies maintained higher levels of carbohydrates with ageing. Therefore, 120QHTT^ex1^ flies displayed an accelerated decline in the systemic levels of carbohydrates relative to body weight and the decline was consistent with the gradual weight loss.

### Abnormal levels of water and protein in 120QHTT^ex1^ flies

**Figure 4:**
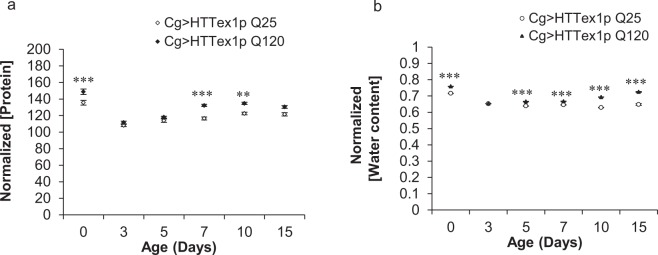
120QHTT^ex1^ adults display altered levels of protein and water content. At the indicated ages, total protein and water content was measured for 25QHTT^ex1^ and 120QHTT^ex1^ females and normalized with the body weights of these flies. (**a)** 120QHTT^ex1^ females have higher protein levels than the age-matched 25QHTT^ex1^ at several time points (day0; day7 onwards). (*n* = 20/genotype/age) (**b)** 120QHTT^ex1^ females have a significant higher water content compared to the age-matched 25QHTT^ex1^. (*n* = 50/genotype/age) Values represent mean ± SEM. *P < 0.05; **P < 0.01; ***P < 0.001.

Body weight management is a complex phenomenon. In addition to carbohydrates and lipids, proteins as well as body water content are other important physiological parameters affecting the body weight and metabolic state of the organism. Assessment of the total protein content of 120QHTT^ex1^ and 25QHTT^ex1^ flies revealed that it tends to remain relatively constant, without normalization, throughout the indicated ages. However, relative to body weight, total protein content of 120QHTT^ex1^ flies was found to be higher at several time points in comparison to age-matched 25QHTT^ex1^ (Fig. 4a; two-factor ANOVA; genotype main effect: F(1, 48) = 72.71, *P* < 0.0001; age main effect: F(5, 48) = 64.89, *P* < 0.0001; age × genotype effect: F(5, 48) = 3.306, *P* = 0.0121). Moreover, 120QHTT^ex1^ flies, exhibited a modest, yet significant, increase in internal water content compared to the age-matched 25QHTT^ex1^ (Fig. 4b; two-factor ANOVA; genotype main effect: F(1, 48) = 1260, *P* < 0.0001; age main effect: F(5, 48) = 609.3, *P* < 0.0001; age × genotype effect: F(5, 48) = 112, *P* < 0.0001). Taken together, systemic protein and water content was higher in 120QHTT^ex1^ flies indicating that unlike other energy biomolecules, hydration and protein levels are not specifically declining in this setting.

### mHTT forms inclusions in the FB cells *in vivo*

**Figure 5:**
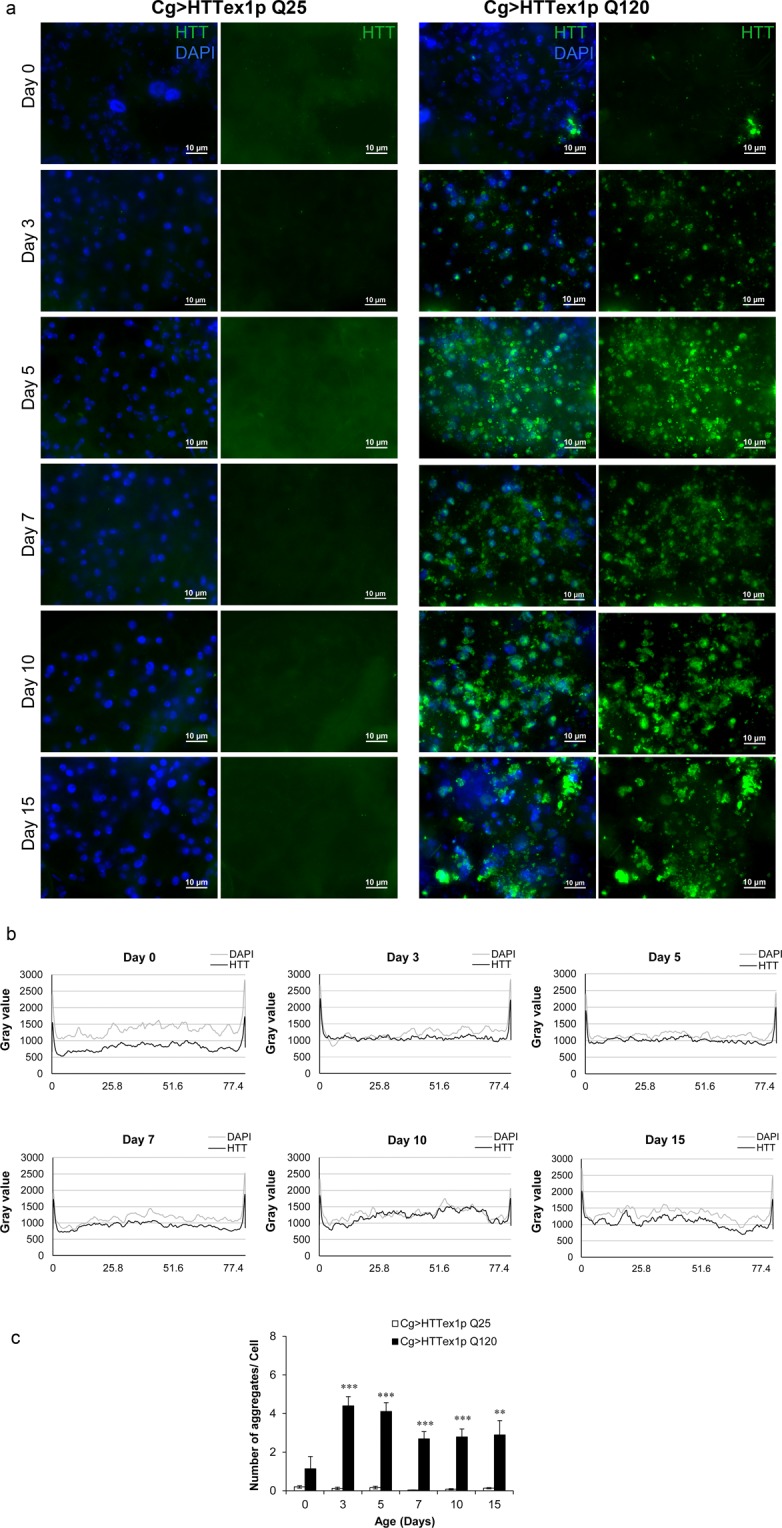
Aggregation of mHTT in the abdominal FB *in vivo*. Abdominal FB dissected from 25QHTT^ex1^ and 120QHTT^ex1^ females at different ages were probed with anti-HTT antibody to reveal the accumulation and/or aggregation of HTT. **(a)** Cg-GAL4 driven-mHTT exon1 peptides form aggregates in the abdominal FB. At day3, aggregates begin to appear as bright puncta (green) in 120QHTT^ex1^ flies (right panel). Along with the aggregates, soluble diffused forms are also detected in the FB at this time point. On the other hand, FB of 25QHTT^ex1^ does not show any accumulation of unexpanded HTT exon1 throughout the indicated ages (left panel). (*n* = 6/genotype/age) Scale bar, 10 µm. **(b)** The image analysis of the FB from 120QHTT^ex1^ flies (a, right panel) is shown as intensity profiles of HTT (black) and DAPI (gray) at different ages. The intensity of HTT signal is lowest at day0 and higher at day10 while the intensity of DAPI signal is relatively constant throughout. **(c)** The number of detectable aggregates per cell was determined by analysing the 3D stacks of all the images in (**a**) using the ImageJ software (*n* = 6/genotype/age). The objects i.e. apparent aggregates are detected strongly in the FB from 120QHTT^ex1^ flies (a, right panel) while the FB from 25QHTT^ex1^ generates negligible signal (a, left panel). The number of objects detected in the day0 old FB from 120QHTT^ex1^ flies specimens is notably less than the number of objects in the following days.

mHTT has been well reported to form intracellular inclusions^63–65^. The formation of intraneuronal inclusions of mHTT and the potential role of mHTT aggregates in HD has been the centre of attention for the last two decades. Hence, to further extend our understanding of these metabolic disturbances occurring in 120QHTT^ex1^ flies relative to mHTT behaviour, we investigated the propensity of mHTT exon1 peptide to form aggregates in the *Drosophila* FB. By immunohistochemistry using N-terminal specific anti-huntingtin antibody (VB3130, human HTT N2–17 specific antibody), the huntingtin-containing inclusions were detected in the abdominal FB cells of 120QHTT^ex1^ adults (Fig. 5a, right panel). In the 25QHTT^ex1^ flies, no accumulation of unexpanded HTT exon1 peptides was observed over time (Fig. 5a, left panel).

In order to determine the natural history of mHTT aggregation, abdominal FBs from 0-, 3-, 5-, 7-, 10- and 15-days old 120QHTT^ex1^ flies was immunostained and analyzed. Interestingly, we found the presence of small yet distinct mHTT aggregates along with amorphous forms at day3 (Fig. 5a, right panel) by the time adult FB is fully developed^66,67^. Further examination at aforementioned ages was done by plotting the intensity profiles of HTT (black) and DAPI (gray) versus position in the FB using NIH ImageJ software (Fig. 5b). The plot profiles revealed that the intensity of HTT signal is relatively low at day0 and much stronger at day10 with the intensity of DAPI signal being comparable at all of the ages. The intensity of HTT signal follows a similar pattern from day3 to day7. At day15, the HTT as well as DAPI signal showed higher fluctuation reflecting lack of tissue in certain regions. Subsequently, to quantify the number of apparent aggregates in the FB, we employed ‘3D Object Counter’ plug-in in the NIH ImageJ software. This analysis strengthened the conclusion that the mHTT exon1 forms aggregates in the abdominal FB of 120QHTT^ex1^ flies (Fig. 5c). The FB isolated from 25QHTT^ex1^ flies displayed negligible objects throughout the indicated ages. The number of aggregates per cell in the FB from day0 old 120QHTT^ex1^ flies was statistically indistinguishable from that of the day0 old 25QHTT^ex1^ FB reinforcing the fact that the adult FB has not yet fully developed. However, at all other ages i.e. day3 to day15, the FB cells of 120QHTT^ex1^ flies exhibited significantly higher number of aggregates compared to the FB cells of age-matched 25QHTT^ex1^ flies (Fig. 5c).

### mHTT induces cell death in the FB of flies

**Figure 6:**
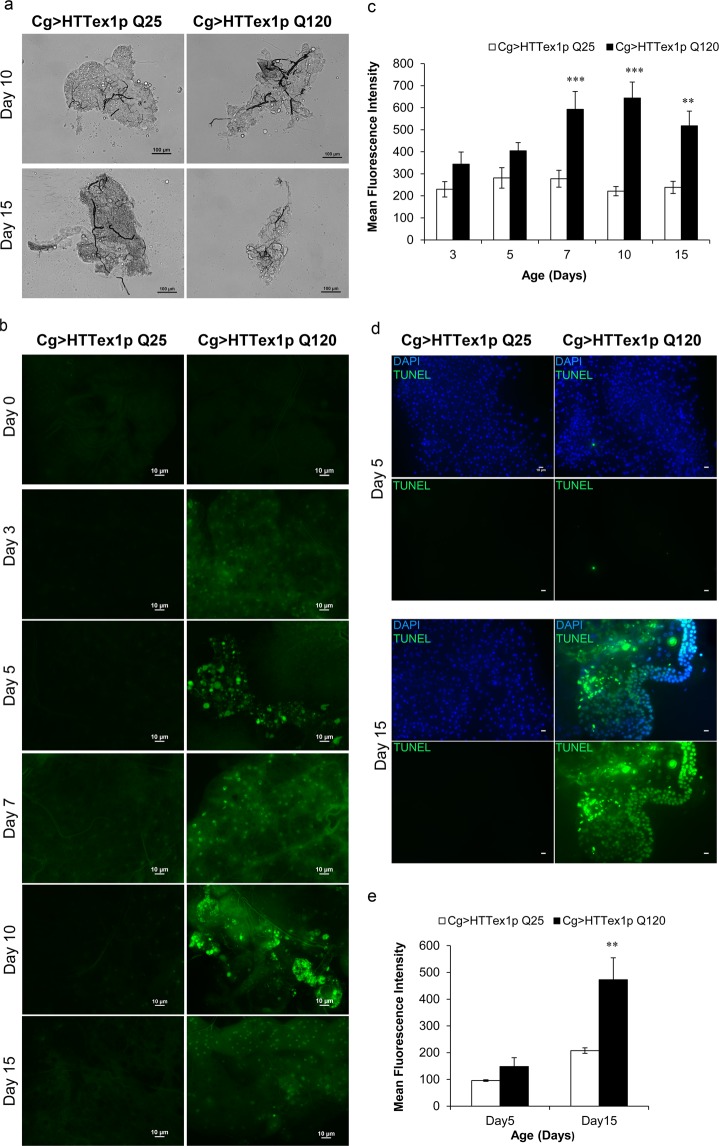
Cell death in the abdominal FB cells of 120QHTT^ex1^ adults. (**a**) Representative bright-field images of abdominal FB from day10 and day15 old 25QHTT^ex1^ and 120QHTT^ex1^ females show dramatically degenerated FB in 120QHTT^ex1^ females. Scale bar, 100 µm. **(b)** Acridine orange (AO) staining was performed on live abdominal FB tissue of both 25QHTT^ex1^ and 120QHTT^ex1^ females throughout the indicated ages (day0-day15). AO positive regions, an indication of cell death, are visible as bright spots (green) of higher intensity throughout the FB in 120QHTT^ex1^ flies. Abdominal FB of 120QHTT^ex1^ flies shows fewer AO positive regions at day5 while a dramatic increase in the proportion of AO positive areas is observed afterwards till day10 of age. At day15, AO signal is slightly reduced than day10 with severe FB degeneration. (*n* = 6–8/genotype/age) Scale bar, 10 µm. **(c)** Histogram representing the mean fluorescence intensity (measured in arbitrary units) of AO in abdominal FB of 25QHTT^ex1^ and 120QHTT^ex1^ females. **(d)** Visualisation of cell death in abdominal FB from 5- and 15- days old 25QHTT^ex1^ and 120QHTT^ex1^ females by TUNEL labelling. TUNEL-positive cells are stained green and nuclei are labelled with DAPI. TUNEL-positive cells are detected in large amounts in the FB of day15 old 120QHTT^ex1^ adults. 25QHTT^ex1^, however, do not show TUNEL-positive cells. (*n* = 8–10/genotype/age) Scale bar, 10 µm. **(e)** Histogram representing the mean intensity of TUNEL signal (green; measured in arbitrary units) in abdominal FB of 25QHTT^ex1^ and 120QHTT^ex1^ females. Values represent mean ± SEM. *** *P* < 0.001; **P < 0.01.

mHTT is reported to cause progressive degeneration and death of neurons majorly in the cortico-striatal circuits^68,69^. Thereby, we further extended our study by examining the fate of FB cells *in vivo*. Surprisingly, microscopic examination of the adult FB tissue of 120QHTT^ex1^ flies revealed clear signs of tissue degeneration (Fig. 6a). Further assessment of the tissue loss was done by acridine orange (AO) staining which specifically labels the apoptotic cells^70^. AO staining revealed prominent cell death in the abdominal FB cells of 120QHTT^ex1^ flies (Fig. 6b). During early ages i.e. up to day3, AO signal was not detected in the abdominal FB cells of 120QHTT^ex1^ flies. However, tissue degeneration along with death of the cells was evident after 5 days posteclosion. Strong AO signal was observed in many regions of the abdominal FB in day7 old 120QHTT^ex1^ flies compared to the age-matched 25QHTT^ex1^ and interestingly, by day10, FB showed higher proportion of AO positive regions implying extensive death in the tissue (Fig. 6c; two-factor ANOVA; genotype main effect: F(1, 51) = 59.59, *P* < 0.0001; age main effect: F(4, 51) = 2.964, *P* = 0.0281; age × genotype effect: F(4, 51) = 3.305, *P* = 0.0175). Along with the evident cell death signal, microscopic examination revealed that the abdomen of 120QHTT^ex1^ flies began to lose the FB by day10 posteclosion (Fig. 6a,b). In addition, TdT-mediated dUTP nick end labelling (TUNEL) of the abdominal FB of 25QHTT^ex1^ and 120QHTT^ex1^ flies also revealed induction of cell death in the FB of 120QHTT^ex1^ flies. A remarkably large proportion of TUNEL-labelled nuclei were detected in the FB of day15 old 120QHTT^ex1^ flies, as compared to the 25QHTT^ex1^ (Fig. 6d,e; day 15, *P* = 0.0090113; Student’s *t* test with unequal variances). In contrast, negligible TUNEL-labelled nuclei were detected in the day5 old FB of 120QHTT^ex1^ flies (Fig. 6d).Taken together, both with AO and TUNEL assay, our results suggest that the accumulation of mHTT in the FB induced cell death *in vivo* possibly via apoptosis.

## Discussion

Unintended weight loss in patients is a complex peripheral manifestation in HD. Moreover, HD patients experience considerable loss of body fat stores^6,10,12,44^ and metabolic abnormalities including high caloric intake^10,12^, altered adipose tissue function^30,31^, and hepatic dysfunction^26–29^. The basis for these peripheral manifestations remains poorly understood. To better understand the basis for weight loss and metabolic abnormalities in HD, we expressed human *HTT* exon1 selectively in the FB of *Drosophila* since the embryonic stage and assessed the detrimental FB-autonomous effects of mHTT exon1 *in vivo*. Notably, it has been reported that the exon1 region of the endogenous *Drosophila* huntingtin protein is least conserved and lacks the polyglutamine and the proline-rich region otherwise present in the human HTT exon1^71^.

We began by characterizing the body weight changes of 120QHTT^ex1^ flies with ageing. We found that 120QHTT^ex1^ flies exhibited gradual weight loss compared to the age-matched 25QHTT^ex1^. Along with the gradual decline in the body weight, 120QHTT^ex1^ flies showed early lethality. Although, 120QHTT^ex1^ females and males displayed similar phenotypes, females exhibited more pronounced decline in their body weight than sibling males and this can be attributed to the sex-specific differences present in the FB of the flies^72,73^. Like humans, where adipose tissue derived hormones act on the hypothalamic nuclei and regulate feeding^74^, *Drosophila* FB also plays a crucial role in sensing the nutritional status of the organism and communicating this information to the neuronal circuitry and in turn, regulates the feeding behaviour^75–77^. Remarkably, we found that food intake was higher in 120QHTT^ex1^ flies compared to the corresponding 25QHTT^ex1^. These flies exhibited a progressive increase in food intake over time and it was inversely correlated with their body weight. Conclusively, despite the increased food consumption, 120QHTT^ex1^ flies were unable to maintain their body weight and resulted in hyperphagic weight loss as seen in HD patients^10,12^. These findings indicate that the selective expression of mHTT in FB is not just affecting the body weight of the flies but a lot more. The weight loss is thus indicative of an insidious metabolic dysfunction culminating in death, which is in line with the chronic weight loss seen in HD patients^5,15^.

*Drosophila* FB plays an indispensable role in energy storage and mobilization. It stores the energy reserves mainly in the form of lipids and glycogen and also synthesizes hemolymph proteins and circulating metabolites. In addition to the energy storage function, FB acts as a nutrient sensor and meets the energy demands of the fly according to the environmental changes^54,55^. In order to uncover the basis behind this toxic weight loss, we assessed the nutrient profile of the 120QHTT^ex1^ flies. We found severe age-dependent decline in the systemic levels of two major biomolecules, lipids and carbohydrates (stored as well as circulating forms). The decline started early during life and worsened with ageing. Moreover, Nile red staining revealed severe depletion of intracellular lipid stores from the abdominal FB cells of 120QHTT^ex1^ flies. Interestingly, systemic decline in the levels of lipids and carbohydrates was in accordance with the chronic weight loss. Nevertheless, the protein content was higher in 120QHTT^ex1^ flies than 25QHTT^ex1^ at several ages. Maintenance of water balance is a crucial physiological phenomenon and any imbalance in the body water levels will also affect the metabolic homeostasis and in turn health of the organism. Here, the 120QHTT^ex1^ flies had higher body water content than 25QHTT^ex1^ of similar ages which could be attributed to the higher feeding in response to insufficient nutrients in the body as when faced with common food/water sources, flies give priority to regulating their nutrient intake via compensatory feeding, at the expense of optimal hydration^78^. Taken together, these findings indicate striking metabolic abnormalities in 120QHTT^ex1^ flies.

In previous studies with R6/2 mice, wasting typically after 11 weeks of age has been reported. Exploration of the mechanism behind this physical wasting in R6/2 mice revealed adipose tissue dysfunction^36,39^. Adipose tissue serves as the major storage depot of lipids as well as plays a key role in the regulation of metabolic homeostasis and in turn body weight majorly through adipokines, namely leptin and adiponectin^79,80^. Interestingly, lipodystrophy, loss of adipose tissue, predisposes patients to insulin resistance, diabetes and leads to a hypermetabolic state^81^. Circulating levels of leptin and adiponectin were found to be significantly decreased in R6/2 mice in later stages. Also, adipose tissue in R6/2 mice exhibited substantial alteration in expression of various adipogenic and lipogenic genes^36^. Moreover, in cultured adipocytes, expression of expanded human huntingtin exon1 (Htt-Q103) had a deleterious effect on the expression of various adipogenic and lipogenic genes^36^. Direct expression of Htt-Q103 in cultured adipocytes also resulted in reduced triglyceride storage within the adipocytes^36^. Another HD mouse model, HD N171, displayed profound thermoregulatory defects and impairment in brown adipose tissue^82^. Interestingly, adipocyte dysfunction has also been suggested in HD patients as when corrected for body fat mass, plasma leptin levels increased significantly with the CAG repeat size and the leptin levels were not correlated with either BMI or fat mass, unlike in controls^31^. In yet another study, HD patients displayed reduced plasma leptin levels indicative of impaired adipose tissue function^30^.

The metabolic alterations occurring in 120QHTT^ex1^ flies may reflect impaired functioning and/or cell death in the FB. To this end, we monitored the fate of FB cells in 120QHTT^ex1^ flies and found substantial cell death occurring in this tissue. Microscopic examination revealed progressive wasting of the tissue and it was found to be consistent with the cell death signal in FB of 120QHTT^ex1^ flies. To our surprise, cell death was most evident at later ages, while metabolic abnormalities started much earlier in these flies. This suggests that besides cell death, functional impairment must be occurring in the FB probably due to the accumulation of mHTT. Indeed, immunohistochemistry analysis of 120QHTT^ex1^ and 25QHTT^ex1^ flies revealed accumulation and aggregation of mHTT exon1 in FB cells of 120QHTT^ex1^ flies. Abdominal FB of day15 old 120QHTT^ex1^ flies was drastically degenerated with large inclusions present in the remaining tissue. In line with our findings, previous studies in various mouse models of HD have shown aggregation in various peripheral tissues as well as in the brain^83,84^. The most plausible hypothesis explaining our results could be that mHTT has direct detrimental effects on the FB cells rendering them functionally impaired initially and eventually leading to FB cell death.

To our knowledge, this is the first *in vivo* report showing that FB-autonomous expression of mHTT exon1 peptide alone is sufficient to cause hyperphagic weight loss along with other metabolic dysfunctions in *Drosophila*. Our results provide a causal link between selective expression of mHTT in FB and chronic weight loss; and might have important implications in understanding peripheral manifestations of HD. If this proves correct and mHTT has such profound effects on adipose tissue and liver in HD patients, then inhibiting the pathological effects of mHTT in these peripheral tissues may be a valuable therapeutic intervention. Nevertheless, given the wide expression of mHTT in HD subjects and the involvement of multiple tissues in the maintenance of adequate body weight^74^, we cannot rule out the implication of other regulatory systems or an indirect signal from neurodegeneration in hypothalamus^85^ in the development of metabolic abnormalities and emaciation in HD. In few mouse model studies, it has been noted that selective expression of mHTT in the hypothalamus could lead to metabolic dysfunctions such as body weight changes and disturbances in the peripheral tissues like brown adipose tissue^35,86^. The available evidence, however, indicates the adipose tissue and liver to be the major determinants of these peripheral manifestations. Further insights into the molecular pathways affected by peripheral mHTT may shed light on the chronic weight loss in HD and might have positive impact on therapeutic interventions aimed at amelioration of HD pathology and improving the quality of life of the patients.

## Materials and Methods

### Fly husbandry

*Drosophila* cultures were reared at 24.5 ± 0.5 °C and 65% humidity on standard cornmeal/sugar/yeast media [6.94 g/L agar (Merck), 41.67 g/L sugar, 16.67 g/L yeast (Merck), 47.23 g/L corn flour, 0.278% propionic acid (Merck), 2.78 g/L nipagin (SDFCL)] under a constant 12 h light: 12 h dark cycle. Expression of human *HTT* exon 1 transgene containing CAG repeats of either 25Qs (wild-type) or 120Qs (mutant) was carried out by using the bipartite UAS-GAL4 expression system in transgenic *Drosophila*. These transgenes are inserted at the same chromosomal location^61^. Transgenic stocks include *w*[*]; M{w[+m*] = UAS-HTTex1p Q25}ZH-51D, *w*[*]; M{w[+m*] = UAS*-*HTTex1p Q120}ZH-51D^61^; kind gifts from J. Lawrence Marsh, UCI, Irvine, California) and fat body specific driver, *collagen*-GAL4 (Cg-GAL4). Virgins of UAS-HTTex1p Q25 and UAS-HTTex1p Q120 were mated with the males of Cg*-*GAL4 and the resulting progeny with 25Qs (Cg-GAL4 > UAS-HTTex1p Q25) represented control and with 120Qs (Cg-GAL4 > UAS-HTTex1p Q120) represented experimental flies.

### Body weight analysis

Adults, both males and virgin females, reared under *ad libitum* fed standard conditions were aged for 0, 3, 5, 7, 10 and 15 days posteclosion and monitored for body weight changes. Whole body weights were measured using Citizen CM11 microbalance and expressed as µg/fly. For body weight analysis, five replicates of 10 flies each per genotype per age were used. (*n* = 50/genotype/age).

### Lifespan assay

Adults, both males and virgin females, of each genotype were collected and were grouped into 20 flies per vial containing standard *Drosophila* food. Flies were maintained as indicated above. Flies were passed to fresh food vials every alternative day and were scored for survival till none remained and represented as Kaplan-Meier survivorship curves. (*n* = 100/genotype).

### Feeding assays

Virgin females were used for all the below mentioned assays conducted in this study. To quantify ad libitum feeding in adult flies, we performed both CAFE assay and blue dye feeding assay. For each assay, flies were habituated on standard *Drosophila* food initially and 5-, 10- and 15-day-old adult females were used. The CAFE assay was slightly modified from the original version^62^ by using standard fly vials containing wet kimwipes as a water source and was soaked in water at regular intervals. A group of 4 females was placed in a vial containing wet kimwipes and two 5 µl micro-capillaries filled with liquid food containing 5% w/v sucrose solution in sterilised distilled water and 1% w/v FD&C blue dye #1. Flies were acclimatized in the CAFE setup for 24 hours before the actual start of the assay in order to familiarize the flies with the new food source. At the start of the assay, old micro-capillaries were replaced with new micro-capillaries and the flies were allowed to feed without any interruption and the feeding was monitored for 24 hours at different ages, namely, day5, day10 and day15. The vials were placed in a chamber along with a water source outside the vials in order to minimize evaporation. At least 15 vials were kept containing micro-capillaries without flies as an evaporation control. For food intake measurements, the amount of liquid food consumed from the experimental micro-capillary was recorded, and the evaporation loss was measured by measuring the change in liquid food volume in the identical vials without flies. We tested the experimental and control flies in each condition simultaneously in order to avoid any variation that could occur due to changing environmental humidity over time. (*n* = 56–65 flies/genotype/age).

For blue dye feeding assay, adult flies were transferred from standard food vial to a vial containing food supplemented with 2% w/v FD&C blue dye #1 and allowed to feed for 2 hours in the morning (ZT 1–3), evening (ZT 8–10) and night (ZT 12–14). After feeding, flies were frozen immediately and then homogenized in 50 µl 1% 1X PBS/Triton X-100 using motorized pestle and centrifuged at 13,500 rpm for 20 minutes. Flies feeding on a standard food vial without blue dye were used as non-fed control and were harvested at same age, homogenized and centrifuged similarly. Absorbance of the supernatant was measured at 627 nm using a NanoDrop 1000 Spectrophotometer and absorbance of the supernatants from flies fed with standard food (typically negligible) was subtracted from the blue dye fed supernatants’ absorbance. The net absorbance was used to calculate the amount of blue dye ingested and the ingested dye concentration of each fly was determined by using the linear fit of the blue dye food standard curve with R^2^ > 0.99^76,87^. The feeding profile for each genotype was determined in at least two independent experiments. (*n* = 50–60 flies/genotype/age).

### Metabolic assays

Adult virgin female flies were analyzed for different biomolecules at the indicated ages, 0, 3, 5, 7, 10 and 15 days. Five replicates of cohorts of four to ten females per genotype per age were taken. All measurements of the biomolecules were normalized to body weight of the respective flies and expressed as µg biomolecules/mg body weight.

#### Lipid quantification

Adult females of a particular age were collected and weighed to obtain whole body weight. Subsequently, the samples were dried in a preheated oven at 70 °C for 36 hours and weighed again to obtain dry body weight. Ether soluble lipids were extracted by transferring intact dried flies to corresponding pre-labelled 1.5 ml microcentrifuge tubes containing 1 ml of diethyl ether at RT on a shaker. The lipids were extracted for 48 hours with three ether changes at an interval of every 12 hours. After the last ether change, flies were dried for 2 hours at 30–35 °C and weighed again to obtain lipid-free weight of the flies. The difference between dry weight and lipid-free weight was considered as the total lipid content of the flies^38^. (*n* = 50/genotype/age).

#### Glycogen quantification

Cohorts of four adult females were homogenized in 400 μl of 2% Na_2_SO_4_. 20 μl of the homogenate was aliquoted and mixed with 46 μl of 2% Na_2_SO_4_ and 934 μl of chloroform/methanol (1:1). The mixture was centrifuged at 13,500 rpm for 10 minutes. 500 μl of anthrone reagent (0.2% anthrone in 72% sulphuric acid) (Sigma Aldrich-319899) was added to the dried pellets and the mixture was heated at 90 °C for 20 minutes^88^. The tubes were then cooled on ice for 10 minutes to stop further reaction and then returned to RT for 20 minutes. The absorbance was recorded at 620 nm. Glycogen concentration was calculated using the linear fit of standard curve with R^2^ > 0.99. (*n* = 20/genotype/age).

#### Trehalose quantification

Cohorts of four adult females were homogenized in 500 μl of 70% ethanol and homogenates were centrifuged at 5,000 rpm for 10 min. at 4 °C to yield pellets. Dried pellets were re-suspended in 200 μl of 2% NaOH, heated at 100 °C for 10 minutes and cooled on ice. 50 μl of the sample was then mixed with 375 μl anthrone reagent and anthrone reaction was performed as indicated above^88^. Absorbance was recorded at 620 nm. Trehalose concentration was calculated using the linear fit of standard curve with R^2^ > 0.99. (*n* = 20/genotype/age).

#### Protein quantification

Cohorts of four adult females were quickly homogenized in 400 μl of 2% Na_2_SO_4_ and 0.05% Tween 20 (1:1). 80 μl of the homogenate was aliquoted and 500 μl of chilled 0.15% sodium deoxycholate was added. The mixture was kept on ice for 10 minutes and 100 μl of 72% trichloroacetic acid was added and incubated again for 15 minutes on ice^89^. The tubes were spun at 8,500 rpm for 15 minutes at 4 °C. Pellet was rinsed once with 1 ml of 1 M HCL, air dried and dissolved in 1.6 ml of cupric sulphate/Bicinchoninic acid solution. The mixture was heated at 60 °C for 10 minutes and tubes were then kept on ice to stop further reaction. Absorbance was recorded at 562 nm. Total protein content was determined using the linear fit of BSA standard curve with R^2^ > 0.99. (*n* = 20/genotype/age).

#### Water quantification

Cohort of 10 female flies were weighed to determine fresh weight and then placed in a preheated oven at 70 °C for 36 hours and reweighed to determine dry weight. Whole-body water content was calculated by the subtraction of mass postdesiccation (dry weight) from its fresh mass (fresh weight) and normalized to body weight of the respective flies and expressed as µg biomolecules/µg body weight. (*n* = 50/genotype/age).

### Lipid droplet staining

Abdomen fillets of minimum five females of same age were dissected out in ice-cold PBS and fixed in 4% formaldehyde/PBT for 20 minutes at RT. After fixation, abdominal FB was detached, fixed for an additional 10 minutes and washed. FB samples were then incubated for 30 minutes in freshly prepared 1:2000 dilution of 0.5 mg/ml Nile Red (Sigma Aldrich- N3013) in PBS at RT. Followed staining, samples were rinsed and mounted in Vectashield with DAPI (Vector Labs). Samples were examined under Nikon Eclipse (Ni-E) fluorescence microscope and lipid droplets quantification in adult FB was done using NIS-Elements AR software. (*n* = 5/genotype/age).

### Immunohistochemistry

Abdominal fillets of adult females of the same age were dissected out in ice-cold PBS and fixed by rocking for 20 minutes at room temperature (RT) in 4% formaldehyde/PBT (PBS + 0.2% Triton X-100). After fixation and blocking in 10% BSA/PBT for 2.5 hours at RT, samples were probed with Anti-Htt antibody (1:5000 dilutions in PBS, Viva Biosciences-VB3130) overnight at 4 °C on a shaker. Abdominal fillets were washed; blocked again for 2.5 hours and probed with secondary antibody (AlexaFluor 488 anti-rabbit at 1:250 in 10% BSA/PBT) for 1.5 hours at RT and washed again. Abdominal FB was then dissected out in PBS and mounted in Vectashield with DAPI. Samples were imaged under Ni-E fluorescence microscope. Image analysis was done using NIS-Elements AR software and ImageJ software. The number of aggregates was counted using ‘3D Object Counter’ plug-in in ImageJ software and the threshold levels were set to 1200. All parameters were kept consistent for all the ages and genotypes. (*n* = 6/genotype/age).

### Cell death assays

#### Acridine orange (AO) assay

AO staining was performed to observe cell death cells in the abdominal FB of adult females at the aforementioned ages. Adult females were dissected in chilled *Drosophila* Ringer’s solution and incubated for 3 minutes in 5 μg/ml AO (Himedia; diluted in Ringer’s solution) in dark^70^. Samples were then washed and imaged immediately using Nikon Eclipse (Ni-E) fluorescent microscope and analyzed with NIS-elements AR software. (*n* = 6–8/genotype/age).

#### TUNEL assay

Abdominal fillets of adult females of the indicated ages were quickly dissected in ice-cold PBS and fixed by rocking for 45 minutes at RT in 4% formaldehyde/PBS. Samples were then washed thrice in 0.1% PBT (1X-PBS + 0.1% Triton X-100) for 5 minutes each, followed by incubation with 1% PBT (20 minutes) and then 75% chilled methanol at 4 °C (15 minutes). The samples were rinsed in PBS and equilibrated at RT for 1 hour, incubated in reaction buffer (TdT enzyme, BrDUTPs and reaction buffer; APO-BRDU kit, Sigma) at 37 °C overnight and afterwards the samples were washed with wash buffer twice for 30 minutes each. Then, samples were rinsed in PBT and blocked in 10% BSA/PBT for 1 hour, followed by incubation with secondary antidigoxigenin antibody for 1.5 hours in dark at RT. Samples were then mounted in Vectashield with DAPI mounting media and analysed. (*n* = 8–10/genotype/age).

### Statistical analysis

All quantified data are presented as mean ± standard error of mean (SEM). Error bars throughout the paper show SEM and the number of subjects is represented by *n*. Normality of the data was determined by Shapiro-Wilk test for normality and variance was determined using Levene’s test. For normally distributed data, analysis was performed using two-factor analysis of variance (ANOVA) followed by Tukey or Bonferroni post-hoc test for multiple comparisons and two-tailed independent Student’s *t* test for pair-wise analysis. All statistical measurements were performed using GraphPad Prism 7 software. Mantel-Cox log-rank test was conducted to obtain statistical significance for survival through OASIS software available at http://sbi.postech.ac.kr/oasis^90^. Significance includes, *P < 0.05, **P < 0.01, ***P < 0.001.

## Supplementary information

Peripheral Expression of Mutant Huntingtin is a Critical Determinant of Weight Loss and Metabolic Disturbances in Huntington’s Disease

## Data Availability

The datasets generated during the current study are available from the corresponding author on request.
