# Supplementary material for: Peripheral Expression of Mutant Huntingtin is a Critical Determinant of Weight Loss and Metabolic Disturbances in Huntington’s Disease
Source: Sci Rep. 2019 Jul 12;9:10127. doi: 10.1038/s41598-019-46470-8 (PMC6626032; doi:10.1038/s41598-019-46470-8)
Supplement: Supplementary file 1 — Peripheral Expression of Mutant Huntingtin is a Critical Determinant of Weight Loss and Metabolic Disturbances in Huntington’s Disease [file 41598_2019_46470_MOESM1_ESM.docx]

**Peripheral Expression of Mutant Huntingtin is a Critical Determinant of Weight Loss and Metabolic Disturbances in Huntington’s Disease**

Priya Lakra^[[1]](#footnote-1)^, Kumari Aditi^1^, and Namita Agrawal^1^ ^,^ ^[[2]](#footnote-2)^*

**Supplementary Figure S1.**

**a**


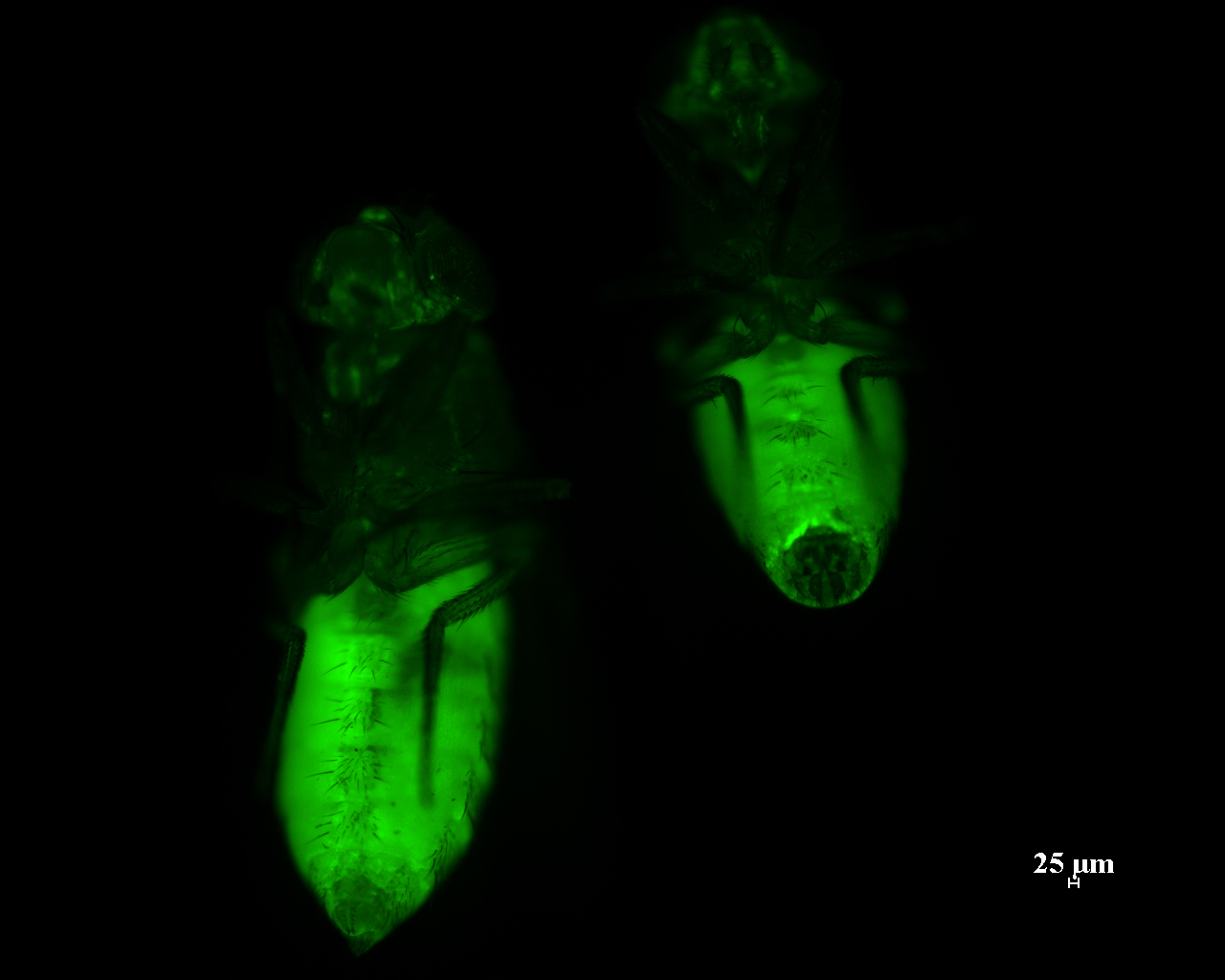

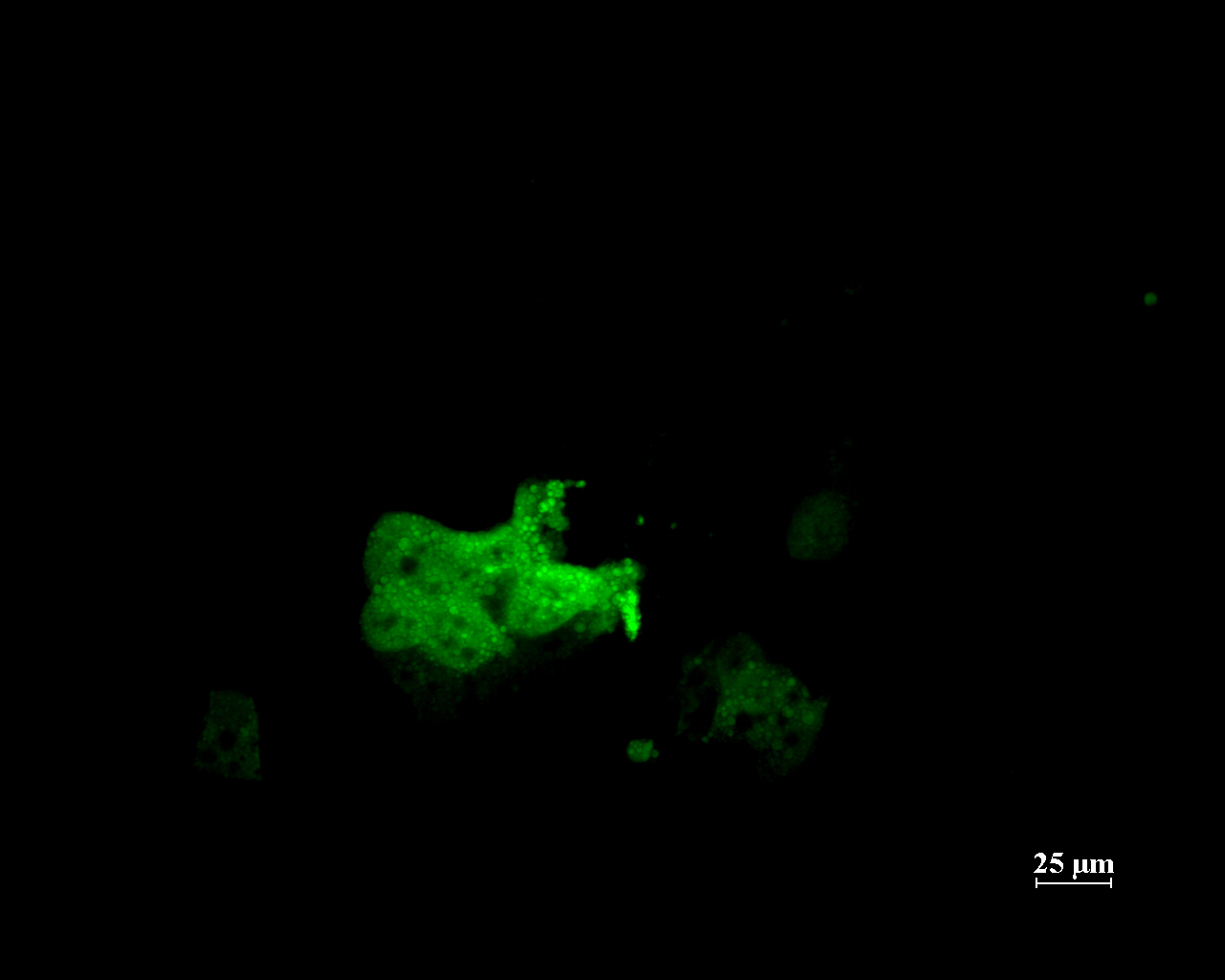


♀

♂

**b**

**Supplementary Figure S1. GAL4-driven GFP expression profile of *Cg* in the adults. (A)** *Cg* is detectable in both female and male *Drosophila* (Cg-GAL4>UAS-Super GFP) and the GFP signal is much stronger in the abdominal region demonstrating FB rich region **(B)** Isolated FB from these adults shows the expression of *Cg* (Green).

**Supplementary Figure S2.**

**a b c**


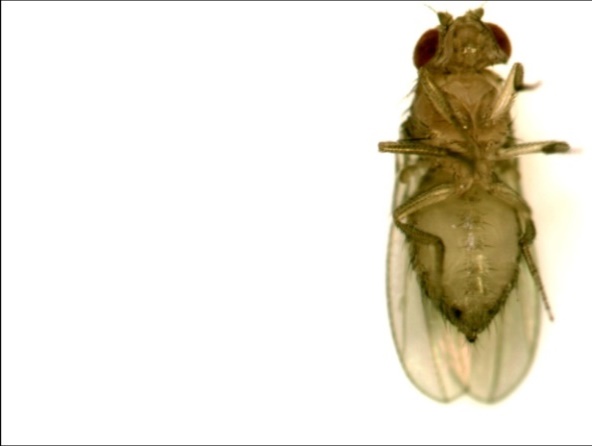

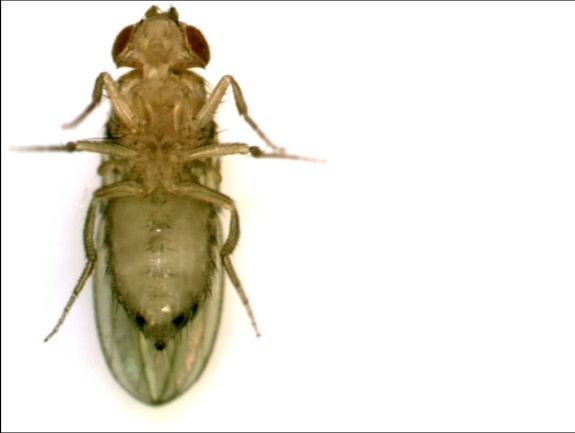

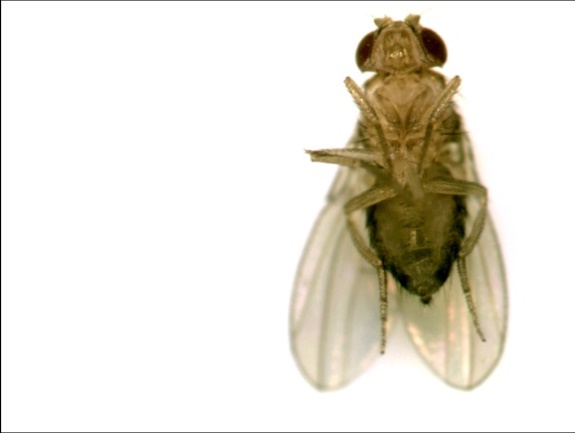


Wild-type 25QHTT^ex1^ 120QHTT^ex1^

**Supplementary Figure S2. FB-autonomous expression of mHTT exon1 causes chronic weight loss in adults. (A, B)** Wild-type *Drosophila melanogaster* and 25QHTT^ex1^ are comparable in phenotype. **(C)** 120QHTT^ex1^ females become phenotypically lean with shrunken abdomen as compared to age-matched wild-type females (a) and 25QHTT^ex1^ females (b) by day15 posteclosion.

1. Department of Zoology, University of Delhi, Delhi - 110007, India [↑](#footnote-ref-1)
2. * Corresponding author. Tel: +91-9717412612; Email: [nagrawaluci@gmail.com](mailto:nagrawaluci@gmail.com) [↑](#footnote-ref-2)
